# Annual acknowledgement of manuscript reviewers

**DOI:** 10.1186/2049-1891-4-4

**Published:** 2013-03-19

**Authors:** Defa Li

**Affiliations:** 1 China Agriculture University, Beijing, China

## Abstract

**Contributing reviewers:**

The editors of *Journal of Animal Science and Biotechnology* would like to thank all our reviewers who have contributed to the journal in Volume 1 (2012).

## 

John Andrae

United States of America

Fuller Bazer

United States of America

Gerd Bobe

United States of America

Eleftherios Bonos

Greece

Martin Braunschweig

Switzerland

Isabelle Cassar-Malek

France

Mingxing Chu

China

Jenifer Cruickshank

United States of America

Cristina Cuello

Spain

Goutam Das

India

Shelly Druyan

Israel

Sandra Edwards

United Kingdom

Gisela F Erf

United States of America

Peter Erickson

United States of America

Wenhai Feng

China

Benjamin Friedrich

Germany

Daniel Gallaher

United States of America

Francisco Gambon-Deza

Spain

Dorian Garrick

United States of America

Rodney Geisert

United States of America

Mohamed Ghanem

Egypt

Laura Giojalas

Argentina

Kevin Glenn

United States of America

Joshua Gong

Canada

Robert Goodband

United States of America

Pawel Gorka

Poland

Kathleen Gough

Canada

Kristen Govoni

United States of America

Agnieszka Grzegorzewska

Poland

Wei Guo

United States of America

PSP Gupta

India

Nagaraja Haleagrahara

Australia

Kazuyoshi Hashizume

Japan

Jung Min Heo

Canada

Gary Hill

United States of America

Alberto Horcada

Spain

Yongqing Hou

China

Walter Hurley

United States of America

Eveline Ibeagha-Awemu

Canada

Ikhide Imumorin

United States of America

Yunliang Jiang

China

Greg Johnson

United States of America

Hiroyuki Kaiya

Japan

David Kenny

Ireland

Jae Cheol Kim

Australia

Changhua Lai

China

Charlotte Lauridsen

Denmark

Hui Li

China

Wen-Wen Lin

Taiwan

Dasen Liu

China

Haiying Liu

China

Wansheng Liu

United States of America

Richard Londraville

United States of America

Carolina Lucci

Brazil

Hailing Luo

China

Juan Marini

United States of America

Gerardo Mariscal Landin

Mexico

Douglas McFarland

United States of America

Kenneth McKeever

United States of America

Jon Meadus

Canada

Sharat Mehta

India

Joris Michiels

Belgium

Subhash Minocha

United States of America

Ronaldo Morato

Brazil

Pascale Mosoni

France

Anita Oberbauer

United States of America

Jack Odle

United States of America

Jie Qiao

China

Velmurugu Ravindran

New Zealand

Renata Ribeiro Alvarenga

Brazil

Johanna Rochester

United States of America

Anshan Shan

China

Jiuzhou Song

United States of America

Dan Stein

United States of America

Michael Sturek

United States of America

Dongxiao Sun

China

Qing-Yuan Sun

China

Lakshmi Sunkara

United States of America

Nosratollah Taherpour Dari

Iran

Alfredo Teixeira

Portugal

Thomas Thymann

Denmark

Stefan Toegel

Austria

Swamy Tripurani

United States of America

Trygve Veum

United States of America

Atte von Wright

Finland

W Walther

Germany

Chunkao Wang

United States of America

Li Wang

China

An-Li Wang

China

Yizhen Wang

China

Malcolm Watford

United States of America

Xin Wei

United States of America

Paul Weimer

United States of America

Guoyao Wu

United States of America

Jingjing Xie

China

Shouqi Xie

China

Gao Xue

China

Huaxiang Yan

China

Caiqiao Zhang

China

Qin Zhang

China

Feng-Qi Zhao

United States of America

Shu-Hong Zhao

China

Huaijun Zhou

United States of America

Xiaoqiu Zhou

China

Shien zhu

China

